# Diversity supplements: An underutilized opportunity to improve the diversity of the health sciences research workforce

**DOI:** 10.1017/cts.2024.21

**Published:** 2024-02-05

**Authors:** Doris Rubio, Noble Maseru, Laura Miller, Mark Geraci, Greg Cooper, Tiffany L. Gary-Webb

**Affiliations:** 1 Institute for Clinical Research Education, University of Pittsburgh School of Medicine, Pittsburgh, PA, USA; 2 Schools of the Health Sciences, University of Pittsburgh, Pittsburgh, PA, USA; 3 Office of Academic Career Development, University of Pittsburgh, Pittsburgh, PA, USA; 4 Office of Research, Health Sciences, University of Pittsburgh, Pittsburgh, PA, USA; 5 Department of Epidemiology, School of Public Health, University of Pittsburgh, Pittsburgh, PA, USA

**Keywords:** Diversity supplements, biomedical research workforce, increase diversity, research training, historically underrepresented groups, infrastructure, social justice

## Abstract

**Purpose:**

This paper describes the process developed at the University of Pittsburgh to increase the number of NIH-funded Diversity Supplements.

**Method:**

The authors formed a Diversity in Academia Workgroup where we created the infrastructure and process to increase the number of Diversity Supplements. Each year, the Office of Sponsored Programs provided a list of grants that would be eligible to submit a Diversity Supplement. We surveyed the Principal Investigators inquiring about their interest in working with a trainee on a Diversity Supplement. If yes, we included their information in a database we built so that trainees could search for eligible research studies. The Diversity Deans then identified underrepresented faculty and postdoctoral researchers. We invited Program Officers from NIH to participate in a panel presentation for trainees, which was well attended.

**Results:**

The number of Diversity Supplements awarded to Pitt researchers has significantly increased from 7 in 2020 to 10 in 2021 and to 15 in 2022. Six more have been awarded in the first half of 2023.

**Conclusions:**

We created the Diversity in Academia Workgroup with the goal to increase the number of Diversity Supplements at the University of Pittsburgh and in so doing, increase the diversity in the biomedical research workforce. While challenging, we know the critical importance and benefits of increased diversity at the University, and we have made significant strides toward this goal.

## Introduction

Diversity on research teams has been shown to increase research productivity and creativity [1], yet the health sciences research workforce still lacks sufficient diversity. Data from the National Science Foundation show that historically underrepresented groups remain underrepresented across the research spectrum; in 2019, only 3% of faculty were Black researchers, 4.7% were Latinos, and 0.2% were Native American [2]. Among the efforts to improve representation is the National Institutes of Health (NIH)’s Research Supplements to Promote Diversity in Health-Related Research Program, which was originally announced in 1989. According to NIH, the purpose of the Diversity Supplements is to “address the need to increase the number of underrepresented minority scientists participating in biomedical research and the health-related sciences [3].” The Diversity Supplements support research experiences for high school students up to and including junior faculty, the intent being that the research exposure will serve as an onramp to a research career. Currently, any active NIH grant with at least 2 years remaining is eligible to apply for a Diversity Supplement [4].

Despite being in existence since 1989, the number of Diversity Supplements is very small compared to the number of R01 grants. Looking at the number of R01 grants from 2005 through 2020, only 2.3% had at least one Diversity Supplement [5]. In 2011, there were only 2 Diversity Supplements. In 2020, there were 455 Diversity Supplements or 4.5% of active R01s. Unfortunately, Diversity Supplements have not led to significant changes in the percent of underrepresented investigators with NIH funding. In 2021, only 2.6% of principal investigators with a research project grant were Black/African American and 4.8% were Hispanic [6].

Given the extent of research being conducted at research-intensive institutions, capitalizing on Diversity Supplements is an opportunity to engage those faculty and trainees who are underrepresented in research. The benefits are bidirectional in that the candidate, or the person applying for the supplement, receives support to engage in research and the research team benefits from diverse perspectives, which has been shown to lead to more productivity, creativity, and innovation and to more relevant and impactful work [7–8].

At the University of Pittsburgh, we formed a Diversity in Academia (DIA) Workgroup in August 2020. The purpose of the DIA Workgroup, which is comprised of senior leaders across the Schools of the Health Sciences, was to increase the representation of researchers from historically underrepresented groups among our institution’s NIH awards and, more specifically, to increase the number of Diversity Supplements. These aims were in support of the broader goals of improving health sciences research workforce diversity and increasing representation of historically underrepresented groups at the University of Pittsburgh.

The decision to initiate the DIA Workgroup was predicated on findings from a white paper produced by the Pitt School of Public Health’s Utilization of Diversity Supplements Work Group, which determined that, in 2016, among the 89 NIH grants at the School of Public Health, only seven (7.8 percent) had funded Diversity Supplements [9].

The formation of the DIA Workgroup also arose in the wake of murders of George Floyd, Breonna Taylor, and Ahmaud Arbery. In response to these tragedies, in June 2020, the University Chancellor, Patrick Gallagher, called on the University community to strengthen their commitment to racial equity and justice. He committed to making the University more diverse, inclusive, and just while also promoting social justice. The Schools of the Health Sciences’ Senior Vice Chancellor, Dr Anantha Shekhar, was one of many to answer the Chancellor’s outcry pledging to make a meaningful contribution to contemporary social justice challenges such as racial equity in the biomedical health sciences workforce. Dr Shekhar, in concert with Provost, Dr Ann Cudd, started a Race and Social Determinants of Equity and Well Being Cluster Hire Initiative designed to significantly increase the hiring, promotion, and retention of faculty from underrepresented groups [10]. To date, the initiative has hired 57 underrepresented faculty over the past three years. The infrastructure established by the DIA Workgroup supports new faculty hired through this initiative since Diversity Supplements are an opportunity for these faculty to obtain support for their research and receive mentorship in their areas of research.

The purpose of this paper is to explain the process we developed over the last three years to increase the number of Diversity Supplements at our institution, which we viewed as a starting point critical scaffolding for creating broader transformational change in recruitment, hiring, retention, mentoring, and faculty development.

## Process

The DIA Workgroup began by working with the Pitt Office of Sponsored Programs to get a baseline of how many Diversity Supplements we had across the institution. The number was even bleaker than that revealed by the School of Public Health’s Work Group: in 2019, of the 709 NIH grants eligible for Diversity Supplements, only seven had funded Diversity Supplements. Seeing that less than 1 percent of eligible grants had a Diversity Supplement affirmed the importance and ramifications this effort has for increasing biomedical research workforce diversity for the University.

**Figure 1.**
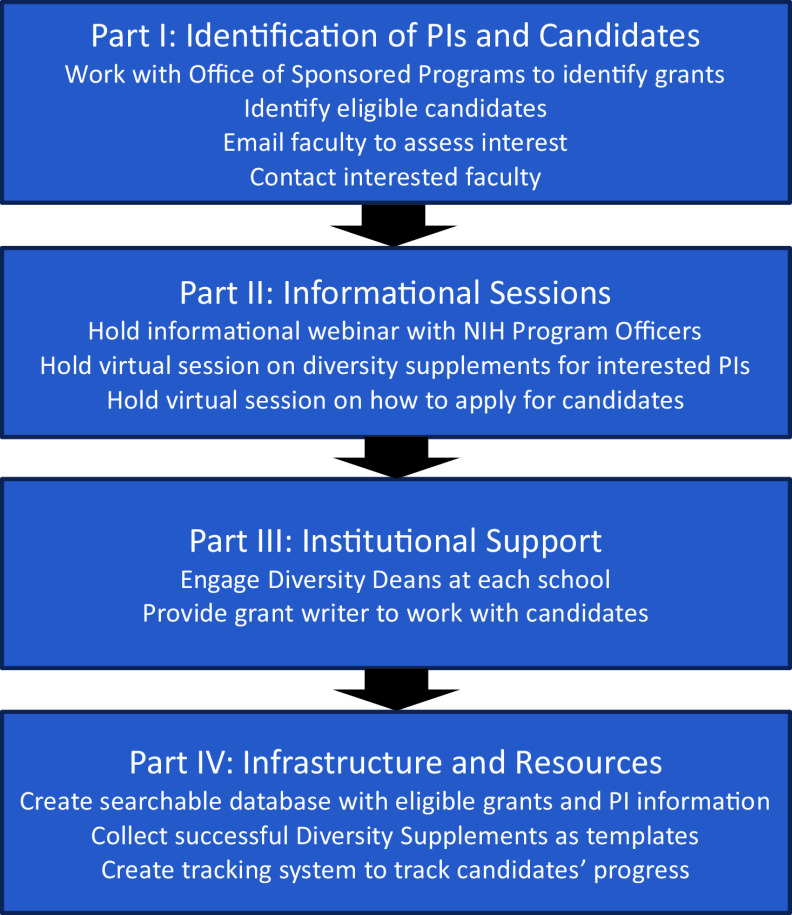
Key elements that led to success.

The DIA Workgroup started with bimonthly meetings to brainstorm ways to set up the infrastructure that would facilitate faculty-candidate pairings and support the development of applications all the way through to submission. We identified the following four key elements (Fig. 1) that in our opinion we would need to be successful: (1) Identification of PIs and candidates; (2) Informational sessions on Diversity Supplements for both candidates and PIs interested in mentoring them; (3) Institutional support, including a grant writer who worked with the candidate and Principal Investigator (PI) through the steps of writing a Diversity Supplement, as well as website development and administrative support; and (4) Infrastructure and resources to help candidates and PIs apply and to track the candidate’s success after submission.

### Identification of PIs and candidates

We took a two-pronged approach by identifying both faculty and candidates who could apply for Diversity Supplements. The Office of Sponsored Programs provided the Office of Research, Health Sciences, with a list of all faculty who had eligible grants with at least two years remaining. The Associate Vice Chancellor for Interdisciplinary Research, Health Sciences, emailed the faculty informing them that they were eligible to apply for a Diversity Supplement on their grant. The email included a question asking if they were: (1) interested; (2) interested but wanted more information; or (3) not interested.

The second part of the two-pronged approach was to identify eligible candidates. This was far more challenging because no central mechanism exists that records people’s underrepresented status. We decided to start with faculty and postdoctoral fellows to make this more manageable as the number of graduate and undergraduate students is considerably larger. We engaged with the Diversity Deans from the Schools of the Health Sciences to create lists of eligible faculty (*n* = 24) and the Office of Academic Career Development to produce a list of underrepresented postdoctoral researchers (*n* = 74), as best as they could identify people based on their records. The Diversity Deans then sent the candidates an email notifying them that: (1) they were eligible to apply for an NIH Diversity Supplement; (2) this initiative was supported by the Office of the Senior Vice Chancellor, Health Sciences; and (3) there was infrastructure to help them identify a laboratory and mentor, develop project aims, and write and submit the Supplement.

Once we had established a process for producing lists of faculty and candidates and then contacting them, we were able to easily repeat the cycle. For example, in each subsequent year, a new list of funded grants eligible for Diversity Supplements was obtained and another email was sent to PIs to identify faculty interested in submitting Diversity Supplements. Those faculty who responded were added to a database on a website developed by the Office of Research, Health Sciences, to house resources related to the DIA Workgroup’s efforts (see #4). The database allows potential candidates to search for grants that would be of interest to them.

### Informational sessions

From the very beginning, we recognized the need to demystify the process of applying for Diversity Supplements. To this end, we held a virtual “How To” session for faculty and trainees. Representatives from NIH, including a Chief from the Office of Programs to Enhance Neuroscience Workforce Diversity and two Program Officers, served as panelists. They each provided an overview of diversity initiatives at their own institutes and explained the specifics and nuances of each. Attendees were then able to ask questions of the panelists. At the end of this main session, NIH representatives offered to go into breakout rooms so that they could meet in small groups with trainees to address their questions more specifically.

Once we had an established infrastructure (see #4) and identified eligible PIs (see #1), we held virtual sessions for PIs and another session for potential candidates. For the PIs, the session consisted of a panel of successful PIs who had a Diversity Supplement as well as one underrepresented mentee who was supported on a Diversity Supplement. The purpose of the panel was for them to explain the process they took and offer any tips for success.

The virtual session developed for potential candidates included an overview of Diversity Supplements as well as breakout rooms tailored for different career stages (undergraduate, graduate, postdoctoral fellows, and junior faculty). Marketing flyers contained a link to the NIH definition for underrepresented populations [11], including those with disabilities and/or from disadvantaged backgrounds, to help increase awareness of who was eligible.

### Institutional support

Very early in the process, we engaged the leaders of the Institute for Clinical Research Education, the Office of the Senior Vice Chancellor, Health Sciences, and the Office for Academic Career Development and shared our goals with them. All the leaders were extremely supportive and offered their commitment and resources to support this work. The DIA Workgroup was provided with administrative support, an experienced grant writer, website development, and resources to track the eligible grants and applications submitted. In addition, the Associate Vice Chancellor for Interdisciplinary Research joined the DIA Workgroup and was the point person for communicating with both the PIs and potential candidates. We also asked the Diversity Deans across the Schools of the Health Sciences to work with the PIs and candidates from their schools.

### Infrastructure and resources

To create an infrastructure that would serve as a self-sustaining process to support these efforts in the future, the DIA Workgroup collaborated with the Office of Research, Health Sciences, who developed a website [12] to house resources for candidates and PIs. More importantly, the website housed a database of eligible grants from PIs who had expressed interest in Diversity Supplements, including links to the detailed NIH RePORTER pages. A dedicated faculty member with expertise in grant writing who works in the Office of Research, Health Sciences, was assigned to work one-on-one with interested candidates to shepherd them and the PI through the process toward submission. We also developed a repository of successful applications (with PIs’ and candidates’ approval) that can be shared with those interested in submitting a Supplement.

The final component in building infrastructure was to create a formal intake and tracking platform. This was done using Microsoft Teams’ “Planner and To Do” mechanism. A Qualtrics survey was used as the intake form, and the results populated the “Planner and To Do” task labeled “New Requests.” From here, DIA Workgroup members can respond to requests, including making notes to track contacts and to advance requests into different categories based on progress. Since the Microsoft Teams group is accessible by all members of the DIA Workgroup, everyone can see the progress of faculty and candidates interested in applying for Diversity Supplements. This ensures that nobody falls through the cracks.

## Results

The DIA Workgroup’s efforts have been successful in terms of heightening the awareness about Diversity Supplements, attendance at informational sessions, the creation of a database of projects and PIs, and increasing the number of successful Diversity Supplement applications. To date, we have offered three informational sessions and workshops, each geared to different audiences. We had a total of 383 people register for those sessions and 224 have attended. The evaluations of the sessions show that attendees found the sessions to be extremely valuable. The sessions have prompted some research faculty to offer their grants for consideration for a Diversity Supplement, and have led multiple trainees to pursue a Diversity Supplement.

We currently have 66 investigators with 78 eligible research projects listed in the database. Each investigator has committed to mentoring an underrepresented candidate to apply for a Diversity Supplement on their grant.

The number of Diversity Supplements awarded to Pitt researchers has significantly increased – from 7 in 2020 to 10 in 2021 and to 15 in 2022. So far in 2023, we have 14 Diversity Supplements, and we expect to have a few more awarded by year’s end.

## Discussion

### Lessons learned – successes and challenges

Overall, our process of engaging faculty and trainees and providing information and support has resulted in a significant increase in successful Diversity Supplement applications across the Schools of the Health Sciences. Having institutional support from senior leadership such as the Senior Vice Chancellor for the Schools of the Health Sciences, as well as a consultative/grant writing resource that could be offered to candidates, were major keys to success. In addition, the DIA Workgroup itself was a key aspect of these efforts, since its members included institutional leaders who hold positions dedicated to the professional development of trainees and faculty, and who are committed to the institutional mission of advancing diversity, equity, and inclusion. In addition, some Workgroup members have considerable expertise in the application process for Diversity Supplements as some were prior applicants and/or served as Diversity Supplement mentors. Further, the consultative support provided to candidates through the workshops, the grant writing consultant, the involvement of Diversity Deans, and the oversight of DIA Workgroup members eliminated many of the barriers to completion that would have existed without those dedicated resources and expertise.

Several challenges arose during the tenure of our Workgroup, which we tried to address swiftly among ourselves and in collaboration with appropriate colleagues. First, the process of identifying candidates, particularly historically underrepresented candidates, was not straightforward. We discovered there was not a university-level process for doing this and that not every school had a systematic process for this. Given the small number of eligible candidates (for example, historically underrepresented Assistant Professors), it was possible to identify candidates through contacts or word of mouth. The DIA Workgroup encouraged each of the Diversity Deans to develop a systematic identification process within their school if there was no process already in place. This was part of our efforts to build long-term Diversity Supplement infrastructure in the Schools of the Health Sciences. Identifying other eligible candidates beyond race/ethnicity, including those with disability status and those who are socioeconomically disadvantaged, also posed a challenge because the data on these categories was not available to the DIA Workgroup. Thus, we heavily emphasized the broad eligibility criteria of the Diversity Supplements during our workshops and conversations so that candidates in those categories would come forward.

Another challenge was the difficulty of providing advice and resources to trainees who were not junior faculty. For example, postdoctoral fellows are a unique group because they are not students, but also not yet faculty members. Therefore, many postdoctoral trainees are at the institution in already funded positions (such as training grants or R01-level grants) and are interested in transitioning to a faculty position. The NIH does not encourage investigators to use the Diversity Supplement mechanism to replace funding already in place for these trainees. Further, there is no built-in mechanism for these trainees to write a supplement as extra support to be considered for a faculty position. Undergraduates are also a group that requires special attention given that their skill level for participating in these grants may be limited and, thus, PIs need to be more directive with planned activities for the trainee in the application.

### Future directions

The DIA Workgroup put considerable effort into developing a process for linking PIs and trainees, giving them support to develop applications, and tracking applications because we believe that this infrastructure is fundamental for improvement and a self-sustaining process. Moreover, additional attention will be given to monitoring the success and trajectory of the trainees. Our workgroup has plans to develop infrastructure around this in addition to the long-term tracking of grant outcomes. Our workgroup will link to existing efforts, such as the Race, and Social Determinants of Equity and Well Being Cluster Hire initiative that was described earlier. As part of the Cluster Hire initiative, historically underrepresented junior faculty participate in career development seminars, retreats, and social outings. There will be opportunities to discuss the benefits and process of applying for Diversity Supplements during these and other similar events.

### Limitations

There are several limitations to our efforts and our reporting of these achievements. Importantly, this examination focuses on the experience of a single institution whose health sciences leadership and faculty are highly committed to workforce diversity, equity, and inclusive excellence. Therefore, our efforts are supported substantially more than they may be at other institutions. Another limitation was the outreach strategies to engage prospective trainees with disabilities and those from disadvantaged backgrounds. Not all trainees disclose their disability, and we have no way to identify those from disadvantaged backgrounds. We continue to advertise this opportunity broadly and hope that trainees self-identify.

## Conclusion

It is challenging to increase the diversity of the biomedical research workforce. This paper describes our efforts to work toward this goal by increasing the Diversity Supplements at our institution and creating an infrastructure to continue supporting this work. With the knowledge that in our health sciences schools, less than 1 percent of NIH-eligible grants acquired a Diversity Supplement, we acknowledged there was clearly a critical need to increase this number for trainees who are underrepresented. Recognizing the underutilization of the opportunity the NIH Diversity Supplement affords, we developed a process to increase grant participation. Our overarching goal was to increase diversity in the biomedical research workforce and, in so doing, to have greater faculty and postdoctoral representation of historically underrepresented groups at the University of Pittsburgh.

Racial equity in health sciences research and in the biomedical workforce can be accomplished by acquiring institutional leadership support and operationalizing the strategic framework presented above. Furthermore, we believe that when the social consciousness of Pitt faculty around equity and social justice is heightened, the value of diversity is realized.
